# Illustrated technique of superficial lymphadenectomy of dogs and cats: preliminary study

**DOI:** 10.29374/2527-2179.bjvm004823

**Published:** 2024-01-17

**Authors:** Matheus Junger do Brasil, Clara Villela Castello Branco, Naomi Cappelli, Joice Bento da Silva, Rosaura Leite Rodrigues, Maria Eduarda dos Santos Lopes Fernandes

**Affiliations:** 1 Undergraduate in Veterinary Medicine, Universidade Castelo Branco, Rio de Janeiro, Rio de Janeiro, RJ, Brazil; 2 Veterinarian, Universidade Estácio De Sá. Vargem Pequena, Rio de Janeiro, RJ, Brazil; 3 Veterinarian, Universidade Castelo Branco, Rio de Janeiro, RJ, Brazil; 4 Veterinarian, MSc., Programa de Pós-graduação em Medicina Veterinária, Departamento de Medicina e Cirurgia Veterinária, Instituto de Veterinária, Universidade Federal Rural do Rio de Janeiro, Seropédica, RJ, Brazil

**Keywords:** lymph node, oncologic surgery, patent blue, linfonodo, cirurgia oncológica, azul patente

## Abstract

Superficial lymphadenectomy is an easy-to-perform and cost-effective routine technique. Despite its simplicity, it remains underutilized in veterinary medicine, with most practitioners being oncological surgeons. This study aims to enhance accessibility to the surgical procedure by providing anatomical representations of superficial lymphadenectomy in the carcasses of dogs and cats. A preliminary study involving two canines and two felines was conducted, with each group comprising a dog and a cat. Group A was designated to superficial lymphadenectomy techniques to create an illustrated step-by-step procedure, while group B underwent anatomical dissection to expose lymph nodes and their adnexa. The approach to superficial lymph nodes in dogs and cats is simple, allowing for the demonstration of superficial lymphadenectomy techniques in the corpses of dogs and cats without complications. This includes the dissection and presentation of anatomical structures adjacent to the lymph nodes. In conclusion, the techniques applied to subjects in groups A and B proved effective, successfully demonstrating and excising all superficial lymph nodes in the corpses of dogs and cats. These findings suggest that the developed set of techniques developed for lymph node excision holds promise for safe and effective application in live animals.

## Resumo

A linfadenectomia superficial é uma técnica de rotina de fácil execução e custo-benefício. Apesar da sua simplicidade, continua subutilizado na medicina veterinária, sendo a maioria dos profissionais cirurgiões oncológicos. Este estudo visa melhorar a acessibilidade ao procedimento cirúrgico, fornecendo representações anatômicas de linfadenectomia superficial em carcaças de cães e gatos. Foi realizado um estudo preliminar envolvendo dois cães e dois gatos, sendo cada grupo composto por um exemplar de cada espécie. O grupo A foi designado para técnicas de linfadenectomia superficial para criar um procedimento passo a passo ilustrado, enquanto o grupo B foi submetido à dissecção anatômica para expor os linfonodos e seus anexos. A abordagem dos linfonodos superficiais em cães e gatos é simples, permitindo a demonstração de técnicas de linfadenectomia superficial em cadáveres de cães e gatos sem complicações. Isto inclui a dissecção e apresentação de estruturas anatômicas adjacentes aos gânglios linfáticos. Concluindo, as técnicas aplicadas aos indivíduos dos grupos A e B mostraram-se eficazes, demonstrando e extirpando com sucesso todos os linfonodos superficiais nos cadáveres de cães e gatos. Essas descobertas sugerem que o conjunto desenvolvido de técnicas para excisão de linfonodos é promissor para aplicação segura e eficaz em animais vivos.

## Introduction

In the field of veterinary medicine, techniques for identifying lymphatic drainage are predominantly employed in the field of veterinary oncology, where they are more commonly practiced (Beserra, 2016; Tuohy et al., 2009). One prevalent method for marking lymphatic pathways in animals involves the application of dyes. Applying intratumoral or peritumoral 2% methylene blue effectively stains lymph nodes in dogs, staining the segment of lymphatic vessels up to the sentinel lymph node in each region (Maués et al., 2016). The technique of lymph node identification using patent blue at a dose of 2 mg/kg has proven effective in identifying and subsequently diagnosing regional metastases in dogs, ensuring proper drainage for the lymph nodes and providing evidence of their location (Estrada et al., 2020).

Although circulation typically refers to the bloodstream, cancer cells have the capacity to disseminate through the lymphatic system, collecting extracellular tissue fluids that ultimately reenter the bloodstream. The drainage of primary tumors is commonly directed to the sentinel lymph node, and evaluating this lymph node and lymphatic basin is important for patient prognosis, survival prediction, and treatment (Grimes et al., 2017; Tuohy et al., 2009). Consequently, the invasion of lymph nodes, serving as primary collection sites for extracellular fluids and debris, is a prevalent occurrence in metastatic processes. The systematic removal and subsequent histopathological evaluation of these lymph nodes are essential and are commonly performed in the diagnosis of tumor metastases (Gonzalez et al., 2016).

Lymphadenectomies can be performed using incisional or excisional techniques (Ehrhart, 2020; Fossum & Caplan, 2014). In veterinary oncology, lymphadenectomy can play an essential role in clinical staging, prognosis determination, treatment plan formulation, and alleviation of tumor burden. Identification and removal of enlarged and abnormal lymph nodes is straightforward, while identification and removal of “normal” peripheral lymph nodes can be challenging. The literature rarely describes techniques for the surgical removal of lymph nodes, with only a few studies and books containing representative images of superficial lymphadenectomy techniques in dogs and cats (Risselada, 2020; Wright & Oblak, 2016).

Despite its inherent simplicity, cost-effectiveness, and ease of execution, superficial lymphadenectomy remains rarely used in routine surgical procedures and is minimally implemented beyond the scope of specialized veterinary oncology (Martins et al., 2020; Wright & Oblak, 2016). Therefore, the primary objective of the present study is to facilitate surgical access and disseminate this procedure through actual anatomical representations, specifically focusing on the surgical technique of superficial lymphadenectomy performed on the carcasses of dogs and cats.

## Material and methods

In the preliminary study, four corpses were used: two canines and two felines. The corpses came from the Veterinária Leopoldina Clinic and were donated by their tutors for this study after the deaths of the animals by signing the term intention to donate the body for teaching purposes. The corpses were kept and refrigerated at -7°C temperature at Veterinária Leopoldina and posteriorly transferred to the anatomopathological laboratory at Universidade Castelo Branco at the same temperature until the present study was conducted.

In Group A, a male mongrel canine with black fur weighing 6 kg and a male mongrel feline with bicolor fur weighing 3 kg were chosen for lymphadenectomy.

In group B, a sixteen-year-old with black and tan dachshund female canine and an approximate eight-year-old female black mongrel feline were selected for lymph node dissection and exposure.

The materials used in this study for gowning were two disposable surgical aprons, a surgical cloak size S, a fenestrated field cloth, two field cloth sizes L, a field cloth size M, a box of procedural latex gloves size M, and surgical masks.

**Figure 1:**
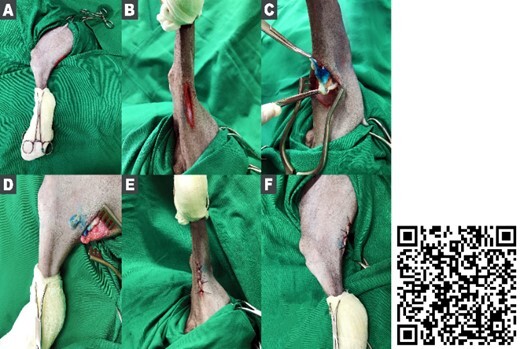
Surgical access for lymphadenectomy of the left popliteal lymph node in a five-month-old mixed breed male canine after injection of 2.5% patent blue (2 mg/Kg) in the distal region of the left pelvic limb. A - The animal was positioned in right lateral decubitus for visualization of the lateral face of the left pelvic limb with a superimposed illustration of the left femoral-tibial-patellar joint.Marking in red, caudal to the knee, shows the site of the surgical incision; B - Caudal aspect of the left pelvic limb after abduction and suspension of the limb, shows the cutaneous surgical incision; C - Divulsed subcutaneous tissue with exposure of the left popliteal lymph node, evidenced by patent blue, located next to the surrounding adipose tissue between the biceps femoris and semitendinosus muscles; D - Left popliteal lymph node removed after performing ligatures of the afferent and efferent vessels; E - Caudal face of the left pelvic limb after abduction and suspension of the limb, showing cutaneous synthesis with standard Sultan *suture*; F - Lateral face of the left pelvic limb shows cutaneous synthesis with standard *Sultan suture*. QR codes directed to *Flyer* contain information on surgical techniques and the topographic dissection of structures attached to popliteal lymph nodes in canines.

Source: Author's collection.

Trichotomy was performed using a Wahl hair clipper machine and a straight razor with eight boxes of Gillette-Wilkinson sword blades. For the application of patent blue were used: four 1 ml syringes, four hypodermic needles (13 X 4.5 mm), and three 2 ml Pharmédice Patent Blue 2.5% ampoules (Figures 1 [Fig gf02]-3).

**Figure 2 gf02:**
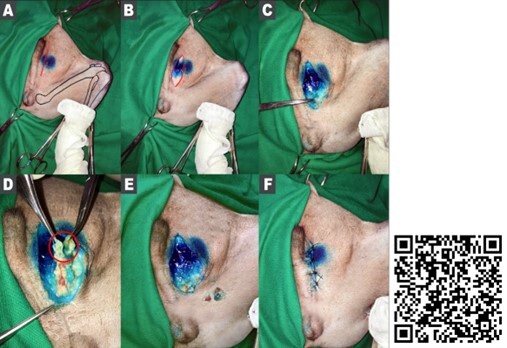
Surgical access for lymphadenectomy of the left inguinal lymph nodes (two) in a five-month-old mixed breed male canine after injection of patent blue 2.5% (2 mg/Kg) in the region of the left inguinal breast (M5). A - The animal was positioned in dorsal decubitus to visualize the medial face of the left pelvic limb and inguinal region, with a superimposed illustration of the left femur and femoral-tibial-patellar joint, and marking in red, between M5 and the penis, showing the site of the surgical incision; B - Cutaneous incision made in the pubic region lateral to the midline, between the M5 and the penis, exposing the subcutaneous cellular tissue; C - Region of location of the left inguinal lymph nodes (tip of the Kelly clamp) after divulsion of the subcutaneous cell tissue; D - Left inguinal lymph nodes (red circles) exposed after divulsion of the subcutaneous cellular tissue; E - Left inguinal lymph nodes removed after ligation of the afferent and efferent vessels; F - Left inguinal region after skin synthesis with standard *Sultan suture*. QR code directed to *Flyer* contains information on the surgical technique and topographic dissection of structures attached to the inguinal lymph nodes in the canines.

Source: Author's collection.

**Figure 3 gf03:**
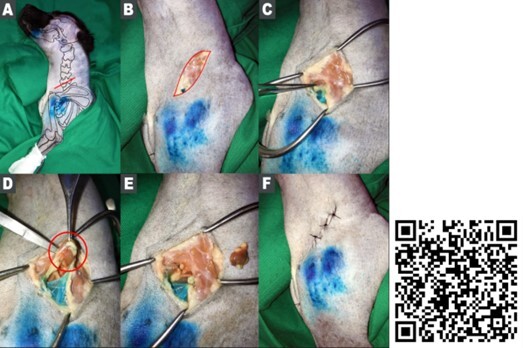
Surgical access for lymphadenectomy of the left superficial cervical lymph node in a five-month-old mixed breed male canine after injection of 2.5% patent blue (2 mg/Kg) in the left cervical-scapular region. A - The animal was positioned in right lateral decubitus with the forelimbs tractioned caudally, neck extended and elevated with a support to visualize the lateral face of the left cranial-cervical-thoracic region with a superimposed illustration of the bones of the region and marking in red, craniodorsal to the left scapulohumeral joint, showing the site of the surgical incision; B - Skin incision made craniodorsal to the left scapulohumeral joint, using the jugular vein as a parameter (dorsal incision to the jugular vein), with subsequent divulsion of the subcutaneous cellular tissue; C - Location of the left superficial cervical lymph node (tip of the anatomical clamp), ventral to the omotransversus muscle, after divulsion of the muscle fibers; D - Left superficial cervical lymph node (red circle) exposed, allowing the identification of its afferent and efferent vessels; E - Left superficial cervical lymph node removed after ligation of afferent and efferent vessels; F - Left cervical-scapular region after synthesis of the omotransversus muscle with simple continuous pattern followed by skin suture with *Sultan pattern*. QR codes directed to *Flyer* contain information on surgical techniques and the topographic dissection of structures attached to the superficial cervical lymph nodes in canines.

Source: Author's collection.

The materials used to perform the lymphadenectomy and dissection techniques were as follows: 2-0 monofilament Nylon box, four tissue pens, four compresses, two Kelly forceps, two Halstead forceps, two Mixter forceps, one Metzenbaum scissor, one Mayo Stille straight scissor, one iris scissor, one Rat Tooth forceps, one Adson forceps, one clinical forceps, four Backaus forceps, two Farabeuf retractors, two Gelpi retractors, one scalpel handle #3, one scalpel handle #4, and scalpel blades # 24 and # 11.

Patent blue was applied with a 1 ml syringe coupled with a hypodermic needle (13 × 4.5 mm) at a dose of 2 mg/kg in the drainage regions of the mandibular, retropharyngeal, superficial cervical, and popliteal lymph nodes, according to Suami et al. (2013). For the axillary and inguinal lymph nodes, the application was performed around the breast for drainage according to Cassali (2018).

The techniques were performed after administering patent blue 2.5% 2 mg/kg), according to a literature review on anatomy and lymphadenectomy. In Group A, surgical superficial lymphadenectomies were performed with the objective of photographing and describing the step-by-step techniques, while in Group B, dissection was performed in the regions of the lymph nodes and surroundings through wide incisions to expose the adjacent structures and their anatomical position, facilitating the recognition of the surgical anatomy.

## Results

Approaching superficial lymph nodes in canines is considered an easy-to-execute technique, allowing the demonstration of lymphadenectomy of all superficial lymph nodes in dogs. The application of 2.5% patent blue proved to be efficient in Group A dogs, even in carcasses, promoting marking through drainage and highlighting in most lymph nodes, except for the axillary and retropharyngeal lymph nodes. The dissection in the canine of group B was a simple execution based on the anatomical literature, making it possible to visualize and highlight structures close to the lymph node, helping to identify and use them as parameters. Patent blue proved ineffective and was unable to mark all lymph nodes. Wide-margin incisions were made for better visualization.

Figures 1 [Fig gf03] [Fig gf04] [Fig gf05]-6 illustrate the step-by-step procedure for performing superficial lymphadenectomy in a dog, representing surgical anatomies through the dissection of superficial lymph nodes (QR Code), and providing illustrated infographics for dissemination in the medical and veterinary academic environment (QR Code).

**Figure 4 gf04:**
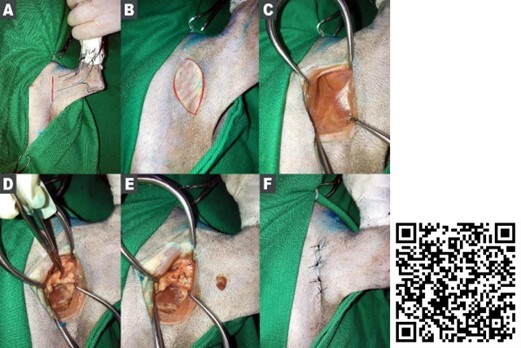
Surgical access for lymphadenectomy of the left axillary lymph node in a five-month-old mixed breed male canine after injection of 2.5% patent blue (2 mg/Kg) in the region of the left axillary breast (M1). A - The animal was positioned in dorsal decubitus with the abduction of the left forelimb, making it possible to visualize the inner face of the left forelimb and axillary region with a superimposed illustration of the humerus-radio-ulnar joint and marking in red, in the middle third of the axillary region, showing the site of the surgical incision; B - Cutaneous incision made in the middle third of the axilla; C - Divulsion of the subcutaneous tissue and subdermal muscles, exposing the pectoral muscles; D - Exposed left axillary lymph node (tip of the anatomical clamp), adjacent to the axillary vein and artery, after divulsion of the deep pectoral muscle; E - Left axillary lymph node removed after ligation of the afferent and efferent vessels; F - Left axillary region after synthesis of the deep pectoral muscle using a simple continuous pattern followed by skin suture using the *Sultan pattern*. The QR code directed to *Flyer* contains information on the surgical technique and topographic dissection of structures attached to axillary lymph nodes in canines.

Source: Author's collection.

**Figure 5 gf05:**
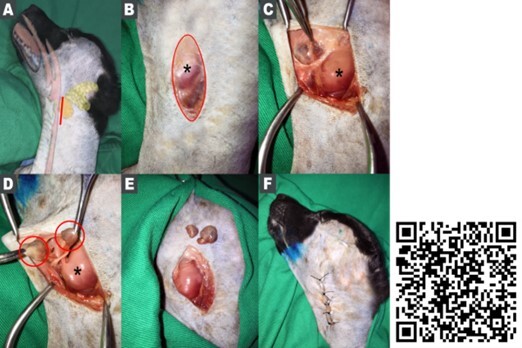
Lateral surgical approach for lymphadenectomy of the left mandibular lymph nodes (two) in a five-month-old mixed breed male canine after injection of 2.5% patent blue (2 mg/Kg) in the region of the left labial commissure. A - The animal was positioned in the right lateral decubitus position with the neck extended and elevated by a support, and forelimbs drawn caudally, making it possible to visualize the lateral face of the cranial-cervical region with a superimposed illustration of the oropharynx, esophagus and trachea and marking in red, caudal to the branch of the mandible, showing the location of the surgical incision; B - Longitudinal skin incision, caudal to the ramus of the mandible, over the left salivary gland (asterisk); C - Region of location of the mandibular lymph nodes (tip of Kelly forceps), cranial to the left mandibular salivary gland (asterisk), after dissection of the subcutaneous tissue, platysma muscle and removal of the paratidal muscle; D - Left mandibular lymph nodes (red circles) exposed, positioned cranially to the left mandibular salivary gland (asterisk); E - Left mandibular lymph nodes removed after ligation of afferent and efferent vessels; F - Cranial-cervical region after synthesis of the platysma muscle using a simple continuous pattern followed by cutaneous suture using the *Sultan* pattern. The QR code directed to *Flyer* contains information on the surgical technique and the topographic dissection of structures attached to the mandibular lymph nodes in canines.

Source: Author's collection.

**Figure 6:**
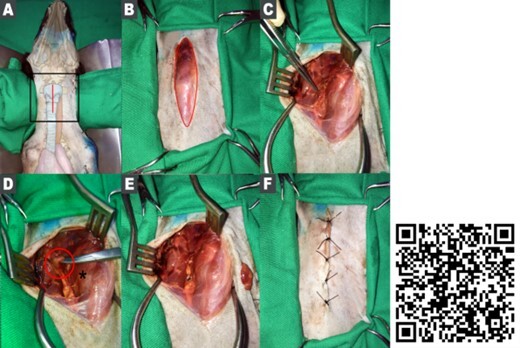
Ventral surgical access for lymphadenectomy of the right retropharyngeal lymph node in a five-month-old mixed breed male canine after injection of 2.5% patent blue (2 mg/Kg) in the region of the labial commissures. A - The animal was positioned in dorsal decubitus with the neck extended and elevated by a support, and thoracic limbs drawn caudally to visualize the ventral-cervical region with a superimposed illustration of the structures of the larynx, and marking in red, on the laryngeal cartilages, showing the location of the surgical incision; B - Median longitudinal skin incision ventral to the larynx; C - Location of the right retropharyngeal lymph node (tip of the anatomical tweezers) after divulsion of the subcutaneous cellular tissue and superficial structures, followed by lateralization of the trachea; D - Right retropharyngeal lymph node (red circle) exposed, positioned caudal medial to the right mandibular salivary gland (asterisk); E - Right retropharyngeal lymph node after ligation of the afferent and efferent vessels; F - Ventral cervical region after cutaneous synthesis with standard *Sultan suture*. QR codes directed to *Flyer* contain information on surgical techniques and the topographic dissection of structures attached to the retropharyngeal lymph nodes in canines.

Source: Author's collection.

Approaching superficial lymph nodes in cats is considered an easily executable technique, facilitating the demonstration of lymphadenectomy for all superficial lymph nodes in cats. The application of 2.5% patent blue was inefficient in the feline in Group A and did not stain any of the removed lymph nodes. The dissection in group B was more difficult due to its state of conservation, proving to be inferior to the other pieces of the study; however, the identification of the structures was easily carried out based on the literature, making it possible to visualize and highlight structures close to the lymph node, helping in its identification and use as a parameter, except for the inguinal lymph node, where it was necessary to use a representation to show it. Patent blue staining was ineffective and did not stain any of the lymphatic structures. Wide-margin incisions were made for better visualization.

**Figure 7:**
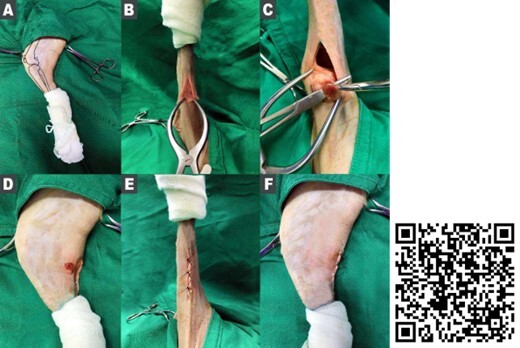
Surgical access for lymphadenectomy of the left popliteal lymph node in a six-year-old mixed breed male feline after injection of 2.5% patent blue (2 mg/Kg) in the distal region of the left pelvic limb. A - The Animal was positioned in the right lateral decubitus position, being possible to visualize the lateral face of the left pelvic limb with a superimposed illustration of the left femoral-tibial-patellar joint and marking in red, caudal to the knee, showing the site of the surgical incision; B - Caudal aspect of the left pelvic limb after abduction and suspension of the limb, showing the cutaneous surgical incision; C - Divulsed subcutaneous tissue with exposure of the left popliteal lymph node located next to the surrounding adipose tissue between the biceps femoris and semitendinosus muscles; D - Left popliteal lymph node removed after performing ligatures of the afferent and efferent vessels; E - Caudal aspect of the left pelvic limb after abduction and suspension of the limb, showing skin synthesis with standard *Sultan suture*; F - Lateral face of the left pelvic limb showing cutaneous synthesis with standard *Sultan suture*. The QR code directed to *Flyer* contains information on the surgical technique and topographic dissection of structures attached to the popliteal lymph node in a feline.

Source: Author's collection.

Figures 7 [Fig gf08] [Fig gf09] [Fig gf10] [Fig gf11]-12 show the step-by-step procedure to perform superficial lymphadenectomy in cats, their respective surgical anatomies represented through the dissection of superficial lymph nodes (QR codes), and illustrated infographics for dissemination in the medical and veterinary academic environment (QR codes).

**Figure 8 gf08:**
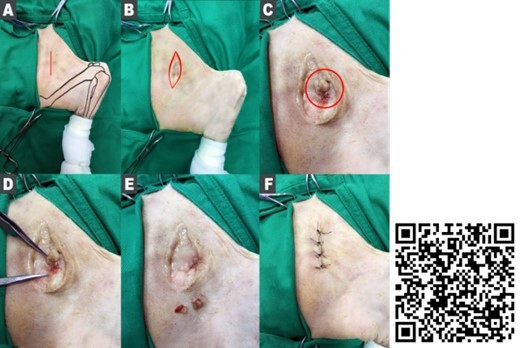
Surgical access for lymphadenectomy of the left inguinal lymph nodes in a six-year-old mixed breed male feline after injection of 2.5% patent blue (2 mg/Kg) in the inguinal breast region (M4). A - The animal was positioned in dorsal decubitus to visualize the medial face of the left pelvic limb and inguinal region, with a superimposed illustration of the femur and left femoral-tibial-patellar joint and marking in red, medial to M4, showing the site of the surgical incision; B - Cutaneous incision made in the pubic region lateral to the midline, medial to M4, exposing the subcutaneous cellular tissue; C - Location of the left inguinal lymph nodes (red circle) after divulsion of the subcutaneous tissue; D - Left inguinal lymph nodes (tip of the anatomical tweezers) exposed after divulsion of the subcutaneous cellular tissue; E - Left inguinal lymph nodes removed after ligation of the afferent and efferent vessels; F - Left inguinal region after skin synthesis with standard *Sultan suture*. QR code directed to *Flyer* contains information on the surgical technique and topographic dissection of structures attached to the inguinal lymph node in a feline.

Source: Author's collection.

**Figure 9 gf09:**
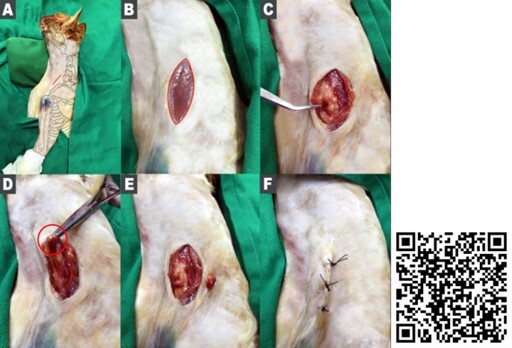
Surgical access for superficial cervical lymph node lymphadenectomy in a six-year-old mixed breed male feline after injection of 2.5% patent blue (2mg/Kg) in the left cervical-scapular region. A - The animal was positioned in right lateral decubitus with the forelimbs tractioned caudally, neck extended and elevated with a support, being possible to visualize the lateral face of the left cranial-cervical-thoracic region with a superimposed illustration of the bones of the region and marking in red, craniodorsal to the left scapulohumeral joint, showing the site of the surgical incision; B - Skin incision made craniodorsal to the left scapulohumeral joint, using the jugular vein as a parameter (dorsal incision to the jugular vein), with subsequent divulsion of the subcutaneous cellular tissue; C - Location of the left superficial cervical lymph node (tip of the anatomical clamp), ventral to the omotransversus muscle, after divulsion of the muscle fibers; D - Left superficial cervical lymph node (red circle) exposed, making it possible to identify its afferent and efferent vessels; E - Left superficial cervical lymph node removed after ligation of afferent and efferent vessels; F - Left cervical-scapular region after synthesis of the omotransversus muscle with simple continuous pattern followed by skin suture with *Sultan pattern*. QR code directed to *Flyer* contains information on the surgical technique and topographic dissection of the structures attached to the superficial cervical lymph nodes in a feline.

Source: Author's collection.

**Figure 10 gf10:**
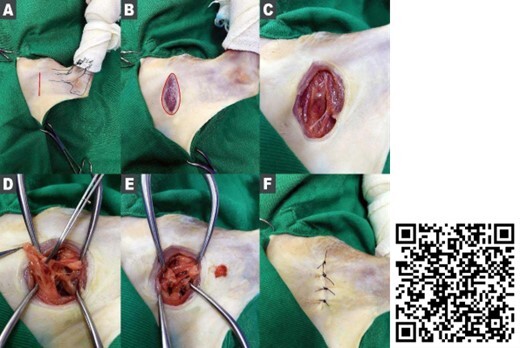
Surgical approach for lymphadenectomy of the left axillary lymph node in a six-year-old mixed breed male feline after injection of 2.5% patent blue (2 mg/mL) in the axillary breast region (M1). A - The animal was positioned in dorsal decubitus with the abduction of the left forelimb, making it possible to visualize the inner face of the left forelimb and axillary region with a superimposed illustration of the humerus-radio-ulnar joint and marking in red, in the middle third of the axillary region, showing the site of the surgical incision; B - Cutaneous incision made in the middle third of the axilla; C - Divulsion of the subcutaneous tissue and subdermal muscles, exposing the pectoral muscles; D - Left axillary lymph node (tip of the anatomical clamp) exposed, adjacent to the axillary vein and artery, after divulsion of the deep pectoral muscle; E - Left axillary lymph node removed after ligation of the afferent and efferent vessels; F - Left axillary region after synthesis of the deep pectoral muscle using a simple continuous pattern followed by skin suture using the *Sultan pattern*. The QR code directed to *Flyer* contains information on the surgical technique and topographic dissection of structures attached to the axillary lymph node in a feline.

Source: Author's collection.

**Figure 11 gf11:**
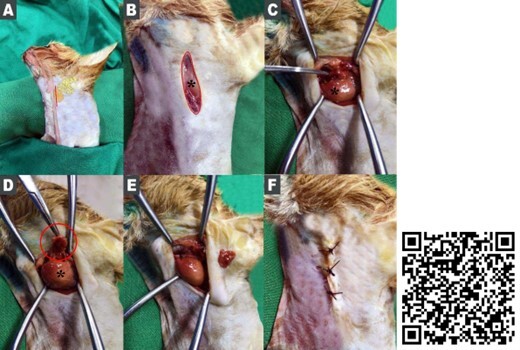
Surgical access for lymphadenectomy of the left mandibular lymph node in a six-year-old mixed breed male feline after injection of patent blue 2.5% (2 mg/Kg) in the region of the left labial commissure. A - The animal was positioned in the right lateral decubitus position with the neck extended and elevated by a support, and forelimbs drawn caudally, making it possible to visualize the lateral face of the cranial-cervical region with a superimposed illustration of the oropharynx, esophagus and trachea and marking in red, caudal to the branch of the mandible, showing the location of the surgical incision; B - Longitudinal skin incision, caudal to the ramus of the mandible, over the left salivary gland (asterisk); C - Location of the mandibular lymph node (tip of the anatomical tweezers), cranial to the left mandibular salivary gland (asterisk), after dissection of the subcutaneous cellular tissue, platysma muscle and removal of the paratidal muscle; D - Left mandibular lymph node (red circle) exposed, positioned cranially to the left mandibular salivary gland (asterisk); E - Left mandibular lymph nodes removed after ligation of afferent and efferent vessels; F - Cranial-cervical region after synthesis of the platysma muscle using a simple continuous pattern followed by cutaneous suture using the *Sulta*n pattern. The QR code directed to *Flyer* contains information on the surgical technique and topographic dissection of structures attached to the mandibular lymph nodes in a feline.

Source: Author's collection.

**Figure 12:**
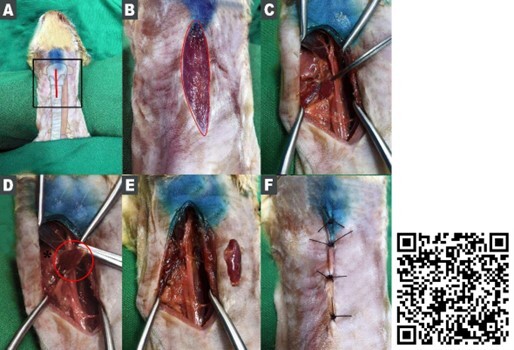
Surgical access for lymphadenectomy of the right retropharyngeal lymph node in a six-year-old mixed breed male feline after injection of 2.5% patent blue (2 mg/mL) in the region of the labial commissures. A - The animal was positioned in dorsal decubitus with the neck extended and elevated by a support, and thoracic limbs drawn caudally to visualize the ventral-cervical region with a superimposed illustration of the structures of the larynx, and marking in red, on the laryngeal cartilages, showing the location the surgical incision; B - Median longitudinal skin incision ventral to the larynx; C - Location of the right retropharyngeal lymph node (tip of the anatomical tweezers) after divulsion of the subcutaneous cellular tissue and superficial structures, followed by lateralization of the trachea; D - Right retropharyngeal lymph node (red circle) exposed, positioned caudal medial to the right mandibular salivary gland (asterisk); E - Right retropharyngeal lymph nodes after ligation of the afferent and efferent vessels; F - Ventral-cervical region after cutaneous synthesis with standard *Sultan suture*. QR code directed to *Flyer* contains information on the surgical technique and topographic dissection of the structures attached to the retropharyngeal lymph nodes in a feline.

Source: Author's collection.

**Figure 13:**
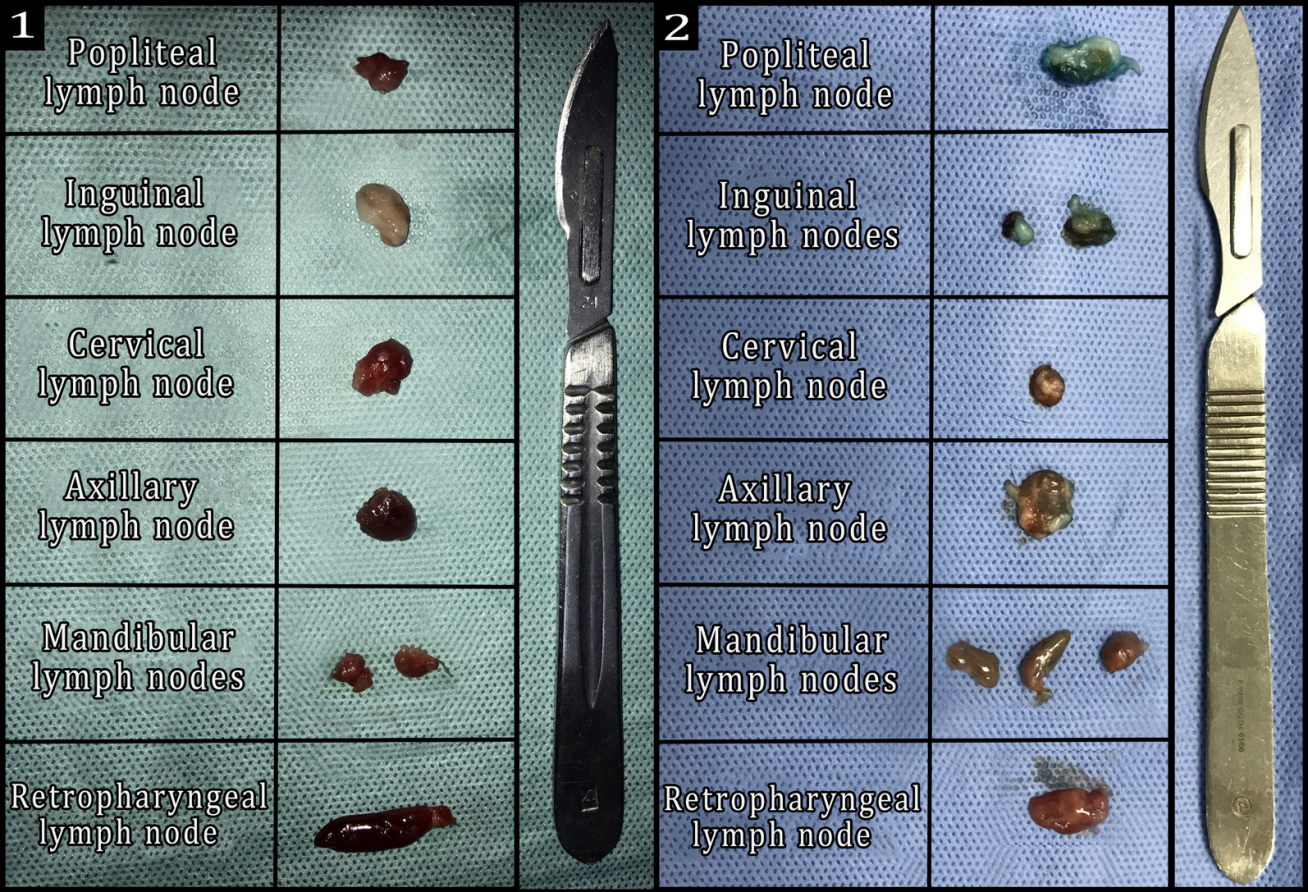
View of the superficial lymph nodes extracted in the dog and cat. 1 - Canine lymph nodes using scalpel handle #4 coupled to blade #24 as a parameter; 2 - Feline lymph nodes using scalpel handle #4 coupled to blade #24 as a parameter.

Source: Author's collection.

Figure 13 shows the superficial lymph nodes of the dog and cat removed after superficial lymphadenectomy.

## Discussion

This study presents a preliminary analysis based on the number of samples used. In this context, a preliminary study serves as an initial step to gain an initial understanding of the lymphadenectomy surgical technique, identify potential trends or patterns, and provide valuable directions for future research. While the small sample size may limit external validity and statistical power, the main objective of this preliminary study was to explore and establish a solid base for further investigation.

The procedures in this study were developed by combining and adapting methods described by Wright and Oblak (2016), Cassali (2018), and Risselada (2020). These methods were simulated on carcasses to create a step-by-step procedure for better understanding and application in clinical surgical routines, demonstrating no difficulty or intercurrence in executing the procedures in the present work.

The positioning of the animal and the incision site for surgical access, following anatomical references described in the literature, proved essential for the successful execution of these techniques. The support used in the cervical region, together with caudally tractioned members, to access the mandibular, retropharyngeal, and superficial cervical lymph nodes was significant, making the lymph nodes evident and providing more effective access (Risselada, 2020; Wright & Oblak, 2016).

The dissection method executed with a wide incision proved its effectiveness in evidencing anatomical structures that help identify the lymph nodes, consistent with the anatomical descriptions found in the literature (Done et al., 2009; Plana et al., 2018). Furthermore, accurate illustrations of the structures near and around the lymph nodes are extremely important for veterinary surgeons to recognize the surgical anatomy and perform lymphadenectomy with greater precision.

In living patients, patent blue and subsequent marking of lymphatic pathways and superficial lymph nodes facilitate the identification of these structures during the lymphadenectomy procedure (Estrada et al., 2020).

Patent blue drainage was shown to be effective; however, this drainage was observed only in some lymph nodes of group A canines. Drainage is expected, followed by highlighting the lymph nodes superior to those observed in this study, even in carcasses where the cardiovascular and circulatory systems are no longer active. The reason for this difference may have been the variation in the time of thawing and performing the lymphadenectomy procedures among the animals, as the surgical technique was executed in the canines of group A 24 h after removal from the freezer, whereas for the other animals, this period was 48 h due to weather conditions.

In this study, we observed that lymph nodes in felines were proportionally larger than those in dogs, corroborating the findings of Done et al. (2009).

Approaches were made to the retropharyngeal and mandibular lymph nodes in canines and felines in Group A through lateral and ventral access according to Wright and Oblak (2016) and Risselada (2020), with the ventral access of the retropharyngeal lymph node proving to be superior for identification and visualization of the lymph node, whereas for the mandibular lymph nodes, identification was easy, both in ventral and lateral access. Therefore, the present study recommends the ventral approach when both lymph nodes need to be removed.

When approaching the superficial cervical lymph node, the need for dissection of the platysma muscle is described to view the omotransversus muscle according to Risselada (2020); however, this muscle was not seen in animals from group A and group B, corroborating other studies that also did not describe the presence of the platysma muscle in this region (Done et al., 2009; Dyce et al., 2010; Konig et al., 2016; Plana et al., 2018).

According to Wright and Oblak (2016), and Risselada (2020), positioning the patient in lateral decubitus with the lateral face of the pelvic limb facing upward to approach the popliteal lymph node, and positioning the patient in lateral decubitus with the pelvic limb suspended and abducted was shown to be more effective, providing better visualization and exposure of the popliteal lymph node.

The identification of the axillary lymph node in animals from groups A and B was demonstrated in all cases close to the thorax, in the region of the rib cage, as reported by Done et al. (2009) and Plana et al. (2018). Therefore, located medially in both dogs and cats. The region where the axillary lymph node is located is composed of important structures such as the nerves of the brachial plexus, veins, and caliber arteries internal to the deep pectoral muscle (Done et al., 2009; Dyce et al., 2010; Konig et al., 2016; Plana et al., 2018). Therefore, we suggest that the location of the axillary lymph node through anatomical references is crucial to avoid affecting these important structures during the identification of the lymph node in question.

Removal of the inguinal lymph node is commonly performed in routine veterinary surgery through mastectomy, in which the entire mammary chain is removed en bloc, with the inguinal lymph node removed simultaneously (Pereira et al., 2019; Petrucci et al., 2021). In this study, it was observed that lymphadenectomy of the inguinal lymph node was easier when performed together with total unilateral mastectomy because of the vast structure removed simultaneously and the proximity of the lymph node and the mammary gland. However, removal of only the inguinal lymph node was more difficult, especially in cats, because of the large amount of fat deposited in the inguinal region. Therefore, strictly following the anatomical references is necessary to successfully perform lymphadenectomy of the inguinal lymph node.

The approach to all canine and feline lymph nodes proved to be simple and easy through the procedures performed, even in carcasses that were deteriorating and in animals without signs of lymphadenopathy. Therefore, an approach without complexity is expected in live animals with lymphadenopathy, where it is often possible to palpate the lymph node to be approached, making surgical access simpler for the surgeon.

The main limitation of this preliminary study was the small number of animals used. However, this study has the potential to offer an initial vision and support future studies that can broaden and deepen our understanding of the subject.

## Conclusion

In this preliminary study, we concluded that the techniques performed on the animals in group A were effective, making it possible to excise all superficial lymph nodes simply and easily, even in carcasses that were deteriorating and in animals without signs of lymphadenopathy. A simple approach is required for live animals and lymphadenopathy. Furthermore, the dissection performed on the animals in Group B provided a better understanding and visualization of the adjacent anatomical structures that helped in the identification of the lymph nodes, making their surgical access even simpler for the surgeon.
